# Difference in axon diameter and myelin thickness between excitatory and inhibitory callosally projecting axons in mice

**DOI:** 10.1093/cercor/bhac329

**Published:** 2022-10-07

**Authors:** Kaustuv Basu, Shailesh Appukuttan, Rohit Manchanda, Attila Sik

**Affiliations:** Facility for Electron Microscopy Research, McGill University, Montreal, QC H3A 0C72, Canada; Department of Anatomy & Cell Biology, McGill University, Montreal, Canada; Biomedical Engineering Group, Department of Biosciences & Bioengineering IIT Bombay, Powai, Mumbay, 4000764, India; Biomedical Engineering Group, Department of Biosciences & Bioengineering IIT Bombay, Powai, Mumbay, 4000764, India; College of Medical and Dental Sciences, University of Birmingham, Vincent Drive, Birmingham B15 2TT, United Kingdom; Institute of Physiology, Medical School, University of Pecs, Pecs H-7624, Hungary; Institute of Transdisciplinary Discoveries, Medical School, University of Pecs, Pecs H-7624, Hungary

**Keywords:** callosal axons, conduction velocity, impulse propagation, myelination, synchronization

## Abstract

Synchronization of network oscillation in spatially distant cortical areas is essential for normal brain activity. Precision in synchronization between hemispheres depends on the axonal conduction velocity, which is determined by physical parameters of the axons involved, including diameter, and extent of myelination. To compare these parameters in long-projecting excitatory and inhibitory axons in the corpus callosum, we used genetically modified mice and virus tracing to separately label CaMKIIα expressing excitatory and GABAergic inhibitory axons. Using electron microscopy analysis, we revealed that (i) the axon diameters of excitatory fibers (myelinated axons) are significantly larger than those of nonmyelinated excitatory axons; (ii) the diameters of bare axons of excitatory myelinated fibers are significantly larger than those of their inhibitory counterparts; and (iii) myelinated excitatory fibers are significantly larger than myelinated inhibitory fibers. Also, the thickness of myelin ensheathing inhibitory axons is significantly greater than for excitatory axons, with the ultrastructure of the myelin around excitatory and inhibitory fibers also differing. We generated a computational model to investigate the functional consequences of these parameter divergences. Our simulations indicate that impulses through inhibitory and excitatory myelinated fibers reach the target almost simultaneously, whereas action potentials conducted by nonmyelinated axons reach target cells with considerable delay.

## Introduction

The formation and recall of sensory, motor, and cognitive representations require coordinated fast communication among multiple cortical areas. The mechanisms underlying long-range coordinated timing of neuronal activity are currently debated. Excitatory principal cells monosynaptically innervate both glutamatergic principal cells and local inhibitory interneurons in each target area and these gamma aminobutyric acid (GABA) expressing inhibitory cells play a key role in the local synchronization of principal cells (Buzsaki 1984; Somogyi et al. 1998). Interneurons temporally coordinate principal neuron activity and modify multiple operations in principal cells via cell domain specific local synaptic innervation (Miles et al. 1996; Somogyi et al. 1998). Studies have shown that cortical GABAergic cells send long-range projections to subcortical and other cortical areas (Alonso and Kohler 1982; Ribak et al. 1986; Leranth and Frotscher 1987; Gonchar et al. 1995; Fabri and Manzoni 1996; Jinno and Kosaka 2002; Fabri and Manzoni 2004; Tomioka et al. 2005). Studies suggest that the corpus callosum could play an inhibitory role (Theory of Inhibition), whereas others say that the corpus callosum serves an excitatory function (Theory of Excitation; Clarke and Zaidel 1994; Bloom and Hynd 2005). The inhibitory model posits that the corpus callosum maintains independent processing between the 2 hemispheres, hindering activity in the opposing hemisphere and causing greater connectivity to increase lateralization (positively correlated; Welcome and Chiarello 2008; Adam and Güntürkün 2009). Lateralization of the brain hemispheres refers to a functional dominance of one hemisphere over the other, in which one is more responsible or entirely responsible for control of a function in comparison with the other (Noggle and Hall 2011). The excitatory model posits that the corpus callosum shares and integrates information between hemispheres, causing greater connectivity to decrease laterality effects by masking the underlying hemispheric differences in tasks that require interhemispheric exchange (negatively correlated; Clarke and Zaidel 1994; Bloom and Hynd 2005; van der Knaap and van der Ham 2011). Ursino and coworkers demonstrated that an excitatory connection to fast inhibitory interneurons is able to transmit the rhythms from one region to the other very efficaciously, whereas connections to slow inhibitory interneurons are less effective (Ursino et al. 2010).

The fiber composition of the corpus callosum has been investigated extensively in number of animal species including rodents, rabbits, cats, monkeys, and humans (Naito et al. 1971; Swadlow 1974; Waxman and Swadlow 1976; Swadlow et al. 1980; Juraska and Kopcik 1988; Gravel and Hawkes 1990; Gravel et al. 1990; Lamantia and Rakic 1990b; Kim et al. 1996; Schüz and Preiβl 1996; Kim and Juraska 1997; Phillips et al. 2015). Cross-species ultrastructural studies addressed callosal fiber diversity including additional species such as cow, horse, and dog (Schüz and Preiβl 1996; Innocenti et al. 1995; Olivares et al. 2001). These studies found that the maximal fiber diameter is higher in species with larger brains, the largest diameter-fibers tend to increase their diameter in larger-brained species, but the interhemispheric transmission velocity does not change with increasing brain size.

However, it is not clear how highly coherent activity, at a precision of milliseconds, is achieved over areas covering hundreds of millimeters by richly branched axons with a wide range of conduction velocities. Studies involving conduction velocity and myelinated nerve fibers started long ago in which more emphasis was given to axoplasmic conductivity and fiber diameter (Sanders and Whitteridge 1946; Rushton 1951; Goldman and Albus 1968; Ritchie 1982). Conduction velocity of myelinated fibers also depends on various physical parameters like internode structure, nodal structure and function, and temperature (Moore et al. 1978). Linear correlations between the myelin cross-sectional area and the circumference of axon in view of variables such as axon size, myelin thickness, and internodal length were also investigated. In early studies to investigate the role of myelin sheath, it was suggested that conduction velocity depends, in the first instance, upon myelin sheath thickness. Myelinated axons (fibers) with thick myelin sheath conduct faster than fibers of similar or even greater diameter but with thinner sheaths (Sanders and Whitteridge 1946). The vast majority of the myelinated axons in the central nervous system (CNS) are very small in diameter (Hildebrand 1971; Franson and Hildebrand 1975; Swadlow et al. 1980; Remahl and Hildebrand 1982; Hildebrand and Waxman 1984). Unmyelinated axons in CNS tracts have diameters up to 0.8 μm. Since these axons lack glial ensheathment they are directly exposed to the extracellular space. Interactions between adjacent axons may lead to subthreshold excitability changes (Kocsis and Waxman 1983). Myelinated CNS axons have diameters down to 0.2 μm, but size spectra of myelinated and unmyelinated axons overlap considerably (Matthews and Duncan 1971; Franson and Hildebrand 1975; Remahl and Hildebrand 1982; Hildebrand and Waxman 1984).

Although previous studies have investigated the fiber diameter composition of the mouse corpus callosum, the existing methodology then did not allow the distinction between excitatory and inhibitory fibers (Innocenti et al. 1995). The purpose of the present study was to investigate the morphological differences, if any, between the long-projecting calcium calmodulin kinase alpha (CamKIIα) immunopositive excitatory axons and the inhibitory axons in the corpus callosum in terms of myelin sheath thickness and axon diameter. The proposal that myelination within the CNS is carried out by oligodendrocytes is now generally accepted and has been demonstrated in electron micrographs (Sturrock 1980). An extensive simulation study was accompanied by morphological measurements to predict possible functional consequences of conduction velocity with regard to differences in structural morphology between the excitatory and inhibitory axons.

## Materials and methods

### Animals

All mice used in the present study were obtained by time-mating wild-type C57BL/6 mice (Charles River Laboratories, Raleigh, NC, United States) with transgenic mice expressing Cre under the CamKIIα promoter (CamKIIα-Cre, Jackson Laboratories; Tsien et al. 1996). Animals were maintained in animal house facilities under a 12-h light/dark cycle in a controlled ambient temperature with normal diet and water ad libitum. Twenty adult (> 5 months) male mice were used for the entire experiment. All experimental procedures were performed in accordance with the guidelines of the Canadian Council of Animal Care and were approved by Laval University Committee on Ethics and Animal Research. All efforts were made to minimize animal suffering and to reduce the number of animals used.

By time-mating wild-type C57BL/6 mice with transgenic mice expressing CamKIIα-Cre mediated recombination, heterozygous mice harboring the Cre recombinase gene as a “knock-in” within its genetic locus was obtained. Mouse tail DNA was used for PCR genotyping. The following primers were used for Cre-genotyping: Cre A F (5′-AGATGTTCGCGATTATC-3′), Cre B R (5′-AGCTACACCAGAGACGG-3′).

### Virus construction

An adenovirus construct was produced by inserting a DNA fragment, CA-promoter (CMV-enhancer+chicken actin-promoter)-loxP-mRFP-loxP-GAP43-palmitoylation-signal -GFP-polyA into SwaI site of pAxcw (Takara Bio, Tokyo, Japan). The adenovirus was produced by transfecting HEK 293 cells with the cosmid DNA. The titer of the adenovirus was raised to 1 × 109 PFU/mL before use by ultracentrifugation. The adenovirus was designated AdlmRFPlGGFP (mRFP; a gift from Dr Roger Chen).

### Tracer injection

BDA injection Biotinylated dextran amine, molecular weight 10,000 (BDA) was dissolved in 0.01-M sodium phosphate buffer at 10% concentration. Micropipettes with tip diameter 20–30 μm were filled with the BDA solution and 0.5 μL was injected into the cortex (coordinates are below).

Virus injection from the virus stock of 1X 109 PFU/mL, 0.5 μL was taken and mixed with 0.5 μL of NaCl (2M) at a ratio of (1:1). We standardized the volume of viral solution to be injected with or without NaCl and found that 0.5 μL of the mixed viral solution with NaCl (1:1) was the best dilution to be administered for each injection. The weights of the mice individually were taken and 0.01 mL of anafen was administered intra-peritoneally before surgery. The animals were anesthetized with 1.4% Isofuran. Adenovirus solution (0.5 μL) was injected into the cortex mediolaterally 3.40 mm, anterior–posterior 0.5 mm and dorsoventrally at 0.5 and 1 mm. After the surgery, the animals were back to full consciousness within a few minutes with normal movement and locomotion. The animals had normal survival periods and none died of viral toxicity or infection.

### Tissue preparation and immunohistochemistry for light microscopy analysis

Animals of the same age (3 weeks old) were injected with tracers. One month after virus injection or 1 week after BDA injection animals were perfused transcardially with 0.9% NaCl, followed by 4% paraformaldehyde in phosphate buffer (pH 7.4). Fixed brains were removed sectioned sagittally at a thickness of 50 μm using a vibrating microtome (Leica, VT1000). After fixations, the tissues were washed again 3 times with PB 0.1 M followed by rinsing 3 × 5 min in Tris Buffer Saline (TBS, 0.0 M, pH 7.4) and blocked for 45 min with 10% normal goat serum (NGS, Vector Laboratories, Burlingame, CA, United States) containing 0.5% Triton X (Sigma-Aldrich, Oakville, ON, Canada). Primary antibodies like rabbit-anti-GFP (1:500, Invitrogen Molecular Probes, Eugene, Oregon, United States), mouse-anti-GFP (1:500, Invitrogen Molecular Probes), mouse-anti-GAD; (1:2,000, Boehringer Mannheim, Germany), mouse-anti-GABA (clone-GB-69,1:100; Sigma), rabbit-anti-RFP (1:2,000, Abcam, United Kingdom), and mouse-anti myelin-associated glycoprotein (MAG; 1:1,000, Chemicon International, Temecula, CA, United States) were diluted in TBS and sections were incubated overnight as the first layer of the immunoreaction. After 3 washes in TBS, sections were incubated for 3 h into either CY2-conjugated goat anti-mouse or CY2-conjugated goat anti-rabbit antibodies to detect GFP (both at 1:500, Jackson ImmunoResearch, West Grove, PA, United States), or CY3-conjugated goat anti-rabbit antibodies (1:500, Jackson ImmunoResearch) to detect RFP, or biotinylated anti-mouse IgG or biotinylated anti-rabbit IgG (both Vector Laboratories) followed by streptavidin-conjugated Alexa 405-conjugated (1:500, Invitrogen Molecular Probes) to detect GAD or GABA. Appropriate controls by omitting primary antisera were performed resulting in no detectable immunosignal except at the injection site (not shown). BDA was detected with streptavidin-conjugated Alexa 546 (1:500, Invitrogen Molecular Probes). Immunoreactions were analyzed under fluorescent (Olympus AX 70) and confocal microscopy (Fluoview-FV1000, Model-1X81, Olympus, United States). FV5-LD405, Ar488, and HeNe1 543 lasers were used to visualize blue, green, and red signals, respectively. To filter suppression of crosstalks, images were taken sequentially in averaged frames using Kalman Filtering.

### Tissue preparation for electron microscopy analysis

Tissue sections were cut sagittally, washed in Tris-buffered saline (TBS, 0.05 M, pH 7.4, Sigma-Aldrich), cryoprotected in 30% sucrose overnight and permeabilized over liquid nitrogen by freeze-thawing. Sections were washed again in TBS and blocked in 5% NGS in TBS for 45 min, then tissues were kept overnight with primary antibodies. The following primary antisera were used: rabbit anti-GFP (1:500, Invitrogen), mouse-anti-GFP (1:500; Invitrogen), and rabbit anti-RFP (1:2,000; Clontech. Lab. Inc.). Sections were thoroughly washed TBS 0.05 M and incubated at room temperature with biotinylated secondary antibody diluted 1:1,000 in TBS for 1.5 h. After washing with TBS tissue were incubated with Avidin biotinylated horseradish peroxidase complex (Elite ABC,1:400, Vector Laboratories) for 1.5 h at room temperature, washed with PB (0.1 M) and the immunoperoxidase reaction was carried out using Ni2 + −intensified 3,3′ diaminobenzidine 4-HCl (DAB, Sigma-Aldrich) as chromogen and 0.03% H2O2. After osmication (1% osmium tetroxide in PB) for 30 min, sections were washed in PB, dehydrated in a graded series of ethanol, counterstained with 1% uranyl acetate in 70% ethanol, cleared in propylene oxide, subjected to overnight infiltration in Durcupan (Fluka, Buchs, Switzerland), flat embedded, and polymerized at 57°C for 48 h. Areas from the anterior (genu) region of the corpus callosum contralateral to the injection site were re-embedded in such a way that the cutting surface was perpendicular to the surface of the sagittal section. Thus, we maximized the chance to sample axons cut across. Serial sections were cut with diamond knife and collected onto Formvar-coated single slot copper grids and counterstained with lead citrate before examination on a Tecnai T12 electron microscope (Thermo Fisher Scientific Inc.) equipped with a Megaview II digital camera (Soft Imaging System, Münster, Germany). For DAB labeling, a non-biotinylated secondary antibody was used (1:1,000) and further developed with Peroxidase anti peroxidase (PAP) conjugated tertiary antibody.

In the sections prepared with the electron microscopy protocol, it was estimated the shrinkage for sections to be symmetrical and 20% in each of the axes; a correction factor of 1.25 was applied to all 3 dimensions for these cells (average of 1.25; Pyapali et al. 1998).

### Data analysis

Diameters of the cross section of axons were measured in *x* and *y* directions on electron microscopy images. To avoid measurement of obliquely cut axons, only those were analyzed whose x/y ratio was between 0.8 and 1.2 representing perpendicular sectioned axons. Both total diameter (axon + myelin sheets = fibers) and axon diameter without myelin were measured.

To determine the difference in axon diameter and thickness of myelination in excitatory and inhibitory axons, first outliers were removed using Outliers labeling rules, then the distribution normality was analyzed with Saphiro–Wilk test, Q–Q plot, and Detrended normal Q–Q plot. Mann–Whitney *U* test after Bonferroni correction and Kruskal–Wallis *H*-test was carried out to analyze differences between axon diameter and myelin thickness of excitatory and inhibitory neurons. Because the group size was often different, significance level was adjusted to avoid sampling bias. Null hypotheses were rejected if the significance level was equal or below 0.05. All statistical analysis was performed using SPSS v21 (IMB, United States) and Microsoft Excel v 2010 (Microsoft, United States).

### Computational model

Computational models of the axons were developed using the compartmental modeling technique on NEURON simulation platform, as illustrated in Fig. 4. Biophysical parameters were set based on a literature survey of findings from experimental studies. Myelination was implemented by means of an increased membrane resistance and reduced membrane capacitance at the internode segments. Capacitance due to myelin sheath was evaluated by considering specific capacitance for an equivalent planar myelin sheath. Nodes of Ranvier were equipped with Hodgkin–Huxley channels, based on those developed for Hippocampal neurons (Traub and Miles 1991), to enable the elicitation of action potentials. Internodes were modeled as possessing only passive membrane channels, and had their membrane properties adjusted corresponding to the extent of myelination. The total membrane capacitance at the internodes was evaluated as a series combination of the capacitance of the cell membrane and the capacitance arising from the myelin ensheathment, and regarding each of these as a cylindrical capacitor. We also compared this approach against treating each as a planar myelin sheath, and arrived at similar results, thereby giving confidence in these parameter values. Action potentials were initiated via current injection at the center of the soma. It was ensured that the lengths of all the axons, myelinated or unmyelinated, inhibitory or excitatory, were always maintained equal for each individual simulation, thereby eliminating such differences as potential causes of simulation outcomes. The internodal length was explored between 5 and 125 μm in our simulations. This range was chosen based on past studies. One such study had reported the variability in the length of internodes, with the shortest being 27 μm and the largest 154 μm (Arancibia-Carcamo et al. 2017). In our model simulations, we found that the action potentials ceased to propagate for internode lengths >125 μm in the case of excitatory myelinated axons, and 150 μm in the case of inhibitory myelinated axons. Tables 1 and 2 provide values for the structural and functional parameters, respectively.

**Table 1:** Structural parameters.

**Parameter**	**Value**
Diameter of soma	25 μm
*Diameter of axon* Excitatory, myelinated Excitatory, nonmyelinated Inhibitory, myelinated Inhibitory, nonmyelinated	679.2 nm 453.8 nm 372.1 nm 334.3 nm
Length of internodes explored	5–125 μm
Mean length of nodes of Ranvier	1 μm
*Mean thickness of myelin lamella* Excitatory Inhibitory	6.8 nm 13.1 nm
*Average number of layers per axon* Excitatory Inhibitory	9.3 7.1

**Table 2:** Functional parameters.

**Parameter**	**Value**
Resting membrane potential: RMP	−70 mV
Membrane capacitance: C_m_	1 μF/cm^2^
Axial (cytoplasmic) resistivity: R_a_	0.1 kΩ.cm
Nernst potential of Na^+^: E_Na_	+50 mV
Nernst potential of K+: E_K_	−77 mV
Internodes—membrane resistance	15 kΩ.cm^2^
Temperature	37°C

## Results

### Contralateral projection of inhibitory neuron in the corpus callosum

**Fig. 1:**
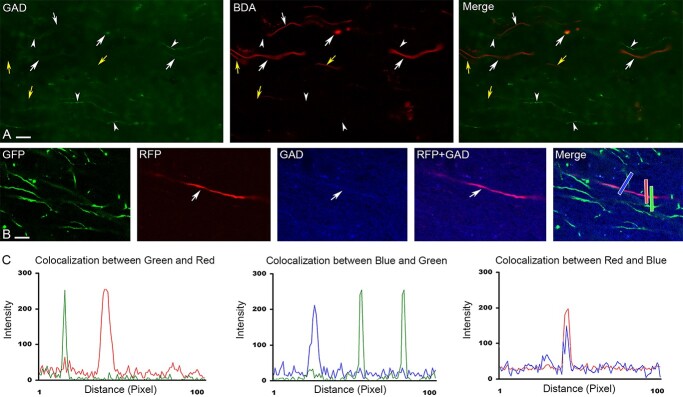
Differential expression of GFP and RFP signals in excitatory and inhibitory axons. A) The tracer BDA labels both inhibitory and excitatory axons in the corpus callosum in the contralateral side of the injection. White arrows show inhibitory (GAD + BDA) long-projection axons, arrowheads depict GAD immunoreactive but unlabeled with BDA axons with unknown origin, whereas yellow arrows show long-projection GAD immunonegative (presumed excitatory) axons. B) GFP is expressed in non-GAD positive (excitatory), whereas RFP is in GAD immunoreactive axons. C) Line profiles indicate that RFP and GFP expression is in separate neuronal profiles (left panel). Middle graph shows that GFP-expressing axons (excitatory) devoid GAD. In contrast, RFP is co-localized with GAD (right panel). Corresponding colored bars on A depicts the measured areas for each graph. Scale: A: 15 μm; B: 10 μm.

We identified the callosally projecting axons by injecting BDA 10k (anterograde tracer, Fig. 1A) and also backfilled cell bodies using retrogradely traveling dye (not shown). We focused our study on the corpus callosum where contralaterally projection axons travel to the contralateral hemisphere. To identify the neurochemical content of the long-projection axons, we double stained the tissue with GAD antibody. A small number of unquantified proportions of the axons were double labeled with the GAD immunoreaction and the tracer material indicating that inhibitory axons cross the midline and innervate the contralateral cortex (Fig. 1A).

### Differential labeling of excitatory and inhibitory axons

Based on their gross morphology excitatory and inhibitory axons cannot be distinguished. Immunohistochemistry can be used to identify inhibitory axons with restrictions because GABA or the synthesizing enzyme GAD is expressed in the axon terminals or in the somata, but myelinated axons segments are immunonegative in most cases as myelin sheath creates a barrier to antibody penetration. To circumvent this problem and differentiate excitatory and inhibitory main axons we used a transgenic mouse line expressing Cre enzyme in CaMKIIa neurons and injected with a virus that expressed GFP in Cre containing (excitatory) cells, whereas Cre negative cells (glial cells and inhibitory neurons) expressed RFP (Basu et al. 2008). Because our aim was to analyze the difference in myelination of long-projection neurons we focused our study on the corpus callosum after unilateral cortical virus injection. We analyzed both the ipsilateral and contralateral sides to the virus injection. Axons in the contralateral side consisted of cross-hemisphere projecting axons. First, using confocal microscopy analysis we determined that there was no overlap between GFP and RFP expression in the virus-infected cells (Fig. 1B). We also demonstrated that on rare occasions when GAD signal was detected in main axons, it was always present in RFP expressing CamKIIα-immunonegative (GABAergic), but not GFP-expressing (excitatory) axons (Fig. 1B and C).

### Ultrastructural study

**Fig. 2:**
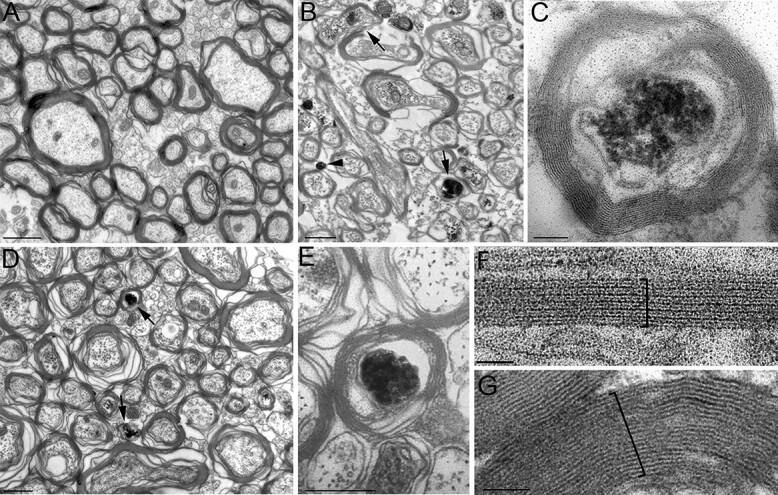
A) The electron microscope picture of sagittally cut sections from unlabeled control corpus callosum show variability in axon diameters and myelin thickness. B) DAB-Ni labeled (electron-dense) myelinated (arrows) and nonmyelinated (arrowheads) excitatory axons have different axon diameter and myelin thickness. C) High-magnification DAB-Ni labeled myelinated excitatory axon. D) DAB-Ni labeled inhibitory axons (arrows) are more scarce and almost always myelinated. E) High-magnification micrograph of myelinated inhibitory axons. F) High-magnification electron microscopy images show the fine structure of myelin sheets of excitatory F) and inhibitory G) projection axons. The number of laminae is more numerous around inhibitory (12 in this example) than excitatory axons (6 in this example). Scale: A, B, D: 500 nm; C: 100 nm; E: 250 nm; F: 40 nm; and G: 80 nm.

The electron microscopy images of sagittally cut sections from the corpus callosum of wild-type noninjected animals showed normal morphology. Most of the axons with varying diameter were myelinated, intermingled with smaller diameter nonmyelinated axons. The axon diameter and myelination were greatly varied (Fig. 2A). To distinguish excitatory axons, we immunostained the GPF-expressing axons in the virus injected transgenic animals using DAB-Ni end-product that results in an electron-dense material that can be easily distinguished in an electron microscope (Fig. 2B and C). We used RFP immunoreaction with the same immunoreaction (DAB-Ni) to visualize inhibitory axons (Fig. 2D and E). In all, 299 myelinated and 413 nonmyelinated excitatory axons were measured in the corpus callosum at the opposite side from the midline of the injection. We have performed similar measurements for the RFP (inhibitory) axons. Because of the scarcity of cross-hemisphere inhibitory projection, we were able to analyze 32 myelinated and 5 nonmyelinated inhibitory axons. To avoid measurement error caused by the inclusion of oblique cut axons that would distort both the diameter measurement and myelin sheath thickness analysis, we measured 2 perpendicular diameters of all labeled axons. We used only the axons for subsequent analysis if the *x* and *y* axonal diameter ratio fell between 0.8 and 1.2. The number of axons that fulfilled the ratio requirement and used for the analysis is indicated in Table 3.

Qualitative inspection suggested that the diameter of excitatory axons was larger than inhibitory and we often observed only thin myelin layers around the excitatory axons. In the case of DAB-Ni labeled inhibitory axons, the myelin layer was thicker. These findings suggested that inhibitory axons had smaller axon diameter and thicker myelin sheath in comparison with excitatory axons.

### Statistical analysis of excitatory and inhibitory axon diameter and myelin thickness

We started the statistical analysis with testing normality. Because most parameters showed non-normal distribution (Table 3), we used corrected nonparametric analysis. First, we compared the diameter of myelinated fibers and nonmyelinated excitatory axons. The axon diameter of myelinated excitatory fibers after subtracting the myelin thickness was significantly larger (679.2 ± 256.1 nm, *n* = 100) than the diameter of nonmyelinated excitatory axons (453.8 ± 183.2 nm, *n* = 159, *P* < 0.001). The diameter of excitatory myelinated fibers were also significantly larger than inhibitory myelinated axons without including myelin sheath thickness (372.1 ± 91.2 nm, *n* = 20, *P* < 0.001). There was no statistical difference between excitatory and inhibitory nonmyelinated axons, nor between the bare axons (myelin thickness subtracted from the diameter of the fiber) of myelinated inhibitory fibers and nonmyelinated inhibitory axons (Table 4).

**Fig. 3:**
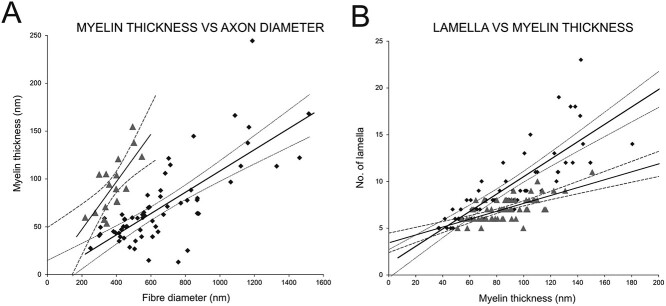
A) Graph showing the relationship between axon diameter and myelin thickness of excitatory (black dots) and inhibitory (gray triangles) long-projection axons. B) The relationship between the myelin thickness and the number of myelin lamellae of inhibitory and excitatory myelinated axons is plotted. Regression lines (solid) with 95% confidence interval (dotted lines) clearly indicate differences in the myelination of excitatory and inhibitory long-projections axons.

Next, we compared the diameter of excitatory and inhibitory myelinated fibers that included the thickness of myelin sheets. Similar to the axons, excitatory myelinated fibers were significantly larger than inhibitory fibers (*P* < 0.001, Table 5, exact *P*-values are not shown). The myelin thickness and number of layers of myelin sheets of excitatory and inhibitory neurons were remarkably different. We used additional high-magnification images to count the number of myelin sheath layers (Fig. 2F and G). The distribution of the thickness of myelin sheath around excitatory and inhibitory fibers was non-normally distributed (excitatory: *P* < 0.00; inhibitory: *P* = 0.025). The thickness of inhibitory fibers myelin sheath was significantly larger than excitatory fibers (*P* < 0.001). The *g* ratio was determined by dividing the diameter of the axon by the diameter of the fiber (axon with myelin). Excitatory fibers had *g* ratio of 0.9 ± 0.027 (*n* = 94) while the *g* ratio value of inhibitory fibers was significantly smaller 0.8 ± 0.034 (*n* = 20; Mann–Whitney *U* test, *P* < 0.001). Interestingly, the myelin thickness of inhibitory neurons increased significantly more with the fiber diameter than the excitatory fibers (Fig. 3A). The number of lamella that made up the myelin ensheathing of the excitatory and inhibitory fibers was also significantly different: on average 9 individual sheets made up the 79-nm thick myelin sheet wrapping excitatory axons, whereas inhibitory axons were ensheathed by 7 lamellae structure making up the 88-nm thick myelin sheath (*P* < 0.001). Unsurprisingly, the thickness of individual lamellae of myelins ensheathing excitatory and inhibitory axons (calculated as thickness of myelin sheath/number of lamella) was also statistically significant (*P* < 0.001; Table 6).

**Table 3:** Diameter of excitatory and inhibitory axons and fibers. The diameters do not include the thickness arising from myelination and correspond to the bare axon.

	***n***	**Distribution**	**Mean (nm)**	**Std Dev**
Excitatory myelinated	100	Non-normal	679.2	256.1
Excitatory nonmyelinated	159	Non-normal	453.8	183.2
Inhibitory myelinated	20	Normal	372.1	91.2
Inhibitory nonmyelinated	3	Normal	334.3	82.8

**Table 4:** Statistical analysis (*P*-values) of axon diameters of long-projection excitatory and inhibitory axons.

	**Excitatory myelinated**	**Excitatory** **nonmyelinated**	**Inhibitory myelinated**	**Inhibitory** **nonmyelinated**
**Excitatory myelinated**	—	0.000	0.000	0.010
**Excitatory nonmyelinated**	0.000	—	0.063	0.226
**Inhibitory myelinated**	0.000	0.063	—	1
**Inhibitory nonmyelinated**	0.010	0.226	1	—

**Table 5:** Myelinated excitatory and inhibitory fiber mean diameters with distribution normality and standard deviation.

	***n***	**Distribution**	**Mean (nm)**	**Std Dev**
**Excitatory myelinated fiber**	100	Non-normal	712.3	292.3
**Inhibitory myelinated fiber**	20	Normal	465.7	113.6

**Table 6:** Thickness, distribution normality, and fine structure myelin ensheathing excitatory and inhibitory axons.

	***n***	**Distribution**	**Mean**	**Std Dev**
**Myelin thickness (excitatory fibers)**	94	Non-normal	63.1 nm	26.6
**Myelin thickness (inhibitory fibers)**	20	Non-normal	93.6 nm	28.4
**Number of wraps** **(excitatory)**	65	Non-normal	9.3	3.8
**Number of wraps** **(inhibitory)**	65	Non-normal	7.1	1.2
**Lamella thickness** **(excitatory)**	95	Non-normal	6.8 nm	2.9
**Lamella thickness** **(inhibitory)**	20	Non-normal	13.1 nm	4.0

To examine whether lamella thickness changes with myelination we plotted the number of lamella that made up the myelin sheath against the myelin thickness. The number of lamella increased significantly more of excitatory neurons than inhibitory fibers, further indicating structural differences of myelin sheets around the 2 analyzed fiber types (Fig. 3B).

### Modeling

Using the values we obtained in the anatomical quantification we constructed a computational model to investigate the functional consequences of the differences between axon diameter, myelin thickness, number of wraps, and lamella structure. Since we were interested in the functional consequences of the difference in condition velocity, we calculated the delay that an action potential would undergo while propagating along the different fibers at various distances: 6 mm taken as average for intra-hemispheric callosal communication, 12 mm as long-distance cross-hemispheric hypothetical connectivity path in rats, and 200 mm as long-distance connection in human brains. The fastest conducting axons were the large diameter excitatory myelinated fibers (0.563 m/s at 12-mm distance) followed by inhibitory fibers (0.551 m/s at 12-mm distance), whereas the nonmyelinated axons conduction velocity was much slower (0.371 m/s for excitatory and 0.317 m/s for inhibitory at 12-mm distance; see Table 7).

**Table 7:** Conduction velocity evaluated for excitatory and inhibitory nonmyelinated and myelinated axons of various lengths from simulations of the model.

	**6 mm**		**12 mm**		**200 mm**	
	**θ (m/s)**	**Δ*t* (ms)**	**θ (m/s)**	**Δ*t* (ms)**	**θ (m/s)**	**Δ*t* (ms)**
**Excitatory myelinated**	0.579	10.3	0.563	21.4	0.550	363.5
**Excitatory nonmyelinated**	0.372	16.0	0.371	32.4	0.370	541.1
**Inhibitory myelinated**	0.561	10.6	0.551	21.8	0.542	369.1
**Inhibitory nonmyelinated**	0.318	18.7	0.317	37.9	0.316	632.7

The simulation outcomes for 6 and 12-mm distances suggest that impulses through inhibitory and excitatory myelinated fibers reach their targets almost simultaneously, whereas action potentials conducted by nonmyelinated axons reach target cells with a larger delay (Table 7). In addition, we also explored for much larger distances of communication to obtain an idea of how these conduction features would extrapolate towards larger brain sizes of humans. Table 7 displays the data thus obtained for the maximal axon length of 200 mm that was investigated. It is seen that the fastest conduction times for such a system in larger brain sizes is ~360 ms, and thus unsuitable for producing interhemispheric synchrony. We discuss the potential implications of this observation below.

We performed a detailed exploration of the effect of the various biophysical parameters on the conduction velocity of the fibers. Each parameter was varied independently, whereas the other parameters were set to their base values as listed in Tables 1 and 2. The parameters were changed within the physiological range observed via our experiments or as reported in literature, and thus were within the scope of biological reality. It was a deliberate choice to vary the parameters between their experimentally reported means and standard deviations, as opposed to uniformly varying them over an arbitrary percentage range, such as ±10%. Our approach gives weight to the different distributions observed experimentally, which are likely to hold physiological significance. For example, functional requirements could necessitate the presence of a larger spread of axonal diameters for excitatory myelinated axons (STD = 277.9 nm) as compared with inhibitory myelinated axons (STD = 91.2 nm).

Figure 5 summarizes the findings from these simulations. Very short axons (~ 2 mm) exhibit comparatively higher conduction velocities owing to boosting from the current stimulus across shorter distances, owing to the well-known phenomenon of reflection of current from the sealed ends of electrically short cables. This quickly vanishes with increasing axonal lengths, and therefore axons of larger lengths typically behave alike. It can be seen that increase in parameters such as axonal diameter, lamella count, and lamella thickness enhance conduction velocity, whereas increase in parameters such as axial resistivity, internodal length, and internodal capacitance lower conduction velocity. It is pertinent to mention that this trend is not entirely monotonic in the case of variations in the internodal length. As seen in Fig. 5E, for very small internodal lengths a positive correlation is observed with respect to conduction velocity. This is in agreement with other theoretical studies in the past (Brill et al. 1977; Moore et al. 1978).

It is notable that proportional variations in the axonal diameters (Fig. 5B), and extent of myelination (Fig 5C and D) has a greater impact on the conduction velocity of excitatory myelinated axons in comparison with inhibitory myelinated axons. Variations in these parameters can therefore easily determine which of these fibers propagate action potentials faster. A similar trend was observed with respect to changes in the internodal length.

Based on the above simulation observations, we performed a sensitivity analysis to evaluate which biophysical parameters affected the conduction velocity most significantly in the myelinated fibers. We also wished to explore whether these parameters could potentially compensate for longer conduction distances in larger sized brains, such as in humans. The parameters that we analyzed are listed in Table 8, and the effect of variation in these parameters was evaluated for axons of varying lengths. The sensitivity was evaluated as the change in conduction velocity to relative change in the biophysical parameter, i.e. slope of the line graph multiplied by 100, where the slope was determined between the 2 closest points on either side of the base value of the parameter employed in the model (see Tables 1 and 2).

We found that the sensitivity to parameters did not alter much with the length of the axons, except for a slight difference for very short axons of ~ 2 mm wherein the response is known to be affected by the applied stimulus, as described previously. Figure 6 shows a comparison of the sensitivity to various biophysical parameters for excitatory and inhibitory myelinated axons of length 12 mm.

**Fig. 4:**
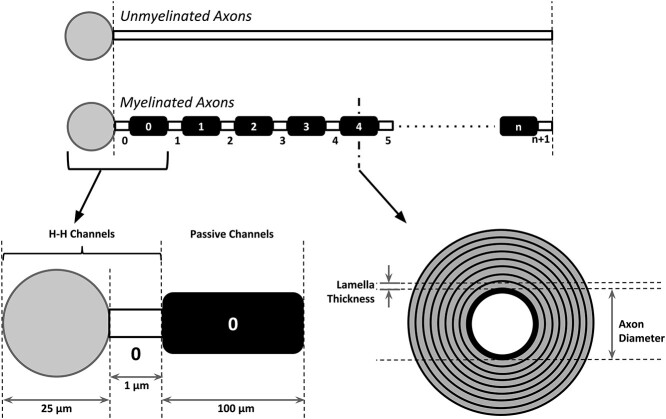
Representation of the models for myelinated and unmyelinated axons. Parameters for the model were sourced from literature and experimental studies. Lengths of all axons were maintained equal for each individual simulation. The sections corresponding to the soma and nodes were incorporated with active channels to enable the elicitation of action potentials. The internodes only contained passive channels and had their membrane properties adjusted corresponding to the extent of myelination.

**Fig. 5:**
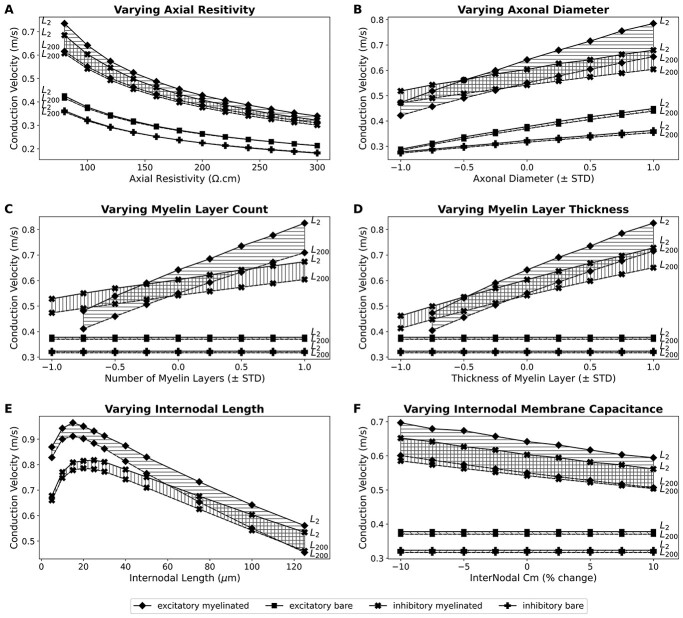
Effect of varying various biophysical parameters on the conduction velocity for axons of various lengths: A) axial resistivity, B) axonal diameter, C) number of layers of myelin, D) thickness of each layer of myelin, E) length of internodes, and F) overall membrane capacitance of internodes. L2 and L200 indicate axons of total length 2 and 200 mm, respectively. Note that for the internodal length a non-monotonic relation is observed, wherein for very short lengths a positive correlation is observed, whereas for larger lengths this trend reverses. In C–F) the unmyelinated fibers do not undergo any changes and are displayed only for ease of comparison with the myelinated fibers.

The sensitivity analysis suggests that changes in internodal capacitance have a notable effect on altering the conduction velocity, as do changes in the extent of myelination. Changes in axonal diameter and axial resistivity have a comparatively moderate influence on the conduction velocity. These are in agreement with previous findings (Moore et al. 1978), except with regard to the effect of internodal length, which is in contrast to that reported earlier. Moore et al. (1978) found the conduction velocity to be relatively insensitive to changes in this parameter. In our study, we found the internodal length to have a strong influence on the conduction velocity, especially for the excitatory myelinated axons. This difference in observations can quite easily arise from the range of internodal lengths explored in each study, and the region of the curve (see Fig. 5E) utilized for analysis. We focused our analysis on the region adjacent to the base value of the parameter (100 μm), where the relation is evidently negative, and concurs with the dominant trend for this parameter.

## Discussion

Explaining the long-range synchronization of cortical activity still remains a major challenge. The present study was undertaken to focus on the mechanisms related to the coordination of cortical impulse activity among the long-range projection neurons, and to find out the functional aspects of conduction velocity between these long-range excitatory and inhibitory neurons in the callosal pathway.

Some functions in the mammalian CNS require the transfer of information between the cerebral hemispheres. A bridge of white matter, the corpus callosum, provides the anatomical substrate for this interhemispheric communication. Although there has been some evidence that inhibitory transcallosal connections exist (Peters et al. 1990; Gonchar et al. 1995; Kimura and Baughman 1997), the general view had been that the interhemispheric connections are largely excitatory (Voigt et al. 1988; Kawaguchi 1992; Conti and Manzoni 1994; Tomioka et al. 2005). Our study provided evidence that GABAergic neurons not only contribute to callosal pathways but also have morphological variations apart from the excitatory in order to conduct impulse in a synchronized way throughout a wide range of areas.

The primate cerebral cortex constitutes a vast communication network of ipsilateral and contralateral corticocortical connections. Although fewer in number, contralateral projection neurons, which course through the corpus callosum and the anterior commissure, have elaborate dendritic trees (Soloway et al. 2002), and are critical for functional integration of the hemispheres reviewed in (Jeeves 1986; Sperry 1986; Trevarthen 1990; Gazzaniga 2000).

The neocortical commissures have a fundamental role in functional integration across the cerebral hemispheres. Barbas et al. investigated whether commissural projections in prefrontal cortices are organized according to the same or different rules as those within the same hemisphere, by quantitatively comparing density, topography, and laminar origin of contralateral and ipsilateral projections, labeled after unilateral injection of retrograde tracers in prefrontal areas. They found that the organization of ipsilateral and contralateral prefrontal projections is similar in topography and relative density, differing only by higher overall density and more widespread laminar origin of ipsilateral than contralateral projections. The projections on both sides are highly correlated with the structural architecture of the linked areas, and their remarkable organization is likely established by punctuated development of distinct cortical types. The preponderance of contralateral projections from layer III may be traced to the late development of the callosal system, whose function may be compromised in diseases that have their root late in ontogeny (Barbas et al. 2005).

Callosal projection neurons interconnect the neocortical hemispheres via the corpus callosum and are implicated in associative integration of multimodal information. Projection neurons have undergone differential evolutionary elaboration, leading to increased diversity of cortical neurons-and more extensive and varied connections in neocortical gray and white matter-in primates compared with rodents. In mouse, distinct sets of genes are enriched in discrete subpopulations of projection neurons, indicating the molecular diversity of rodent projection neurons. Elements of rodent projection neuron functional and organizational diversity might thus be present in the further elaborated primate cortex. To address the hypothesis, Fame et al. found that genes controlling mouse projection neuron subtype diversity might reflect molecular patterns shared among mammals that arose prior to the divergence of rodents and primates. Their studies suggest that there has been evolutionarily differential retraction and elaboration of superficial layer projection neuron subpopulations between mouse and macaque, with independent derivation of novel populations in primates. Together, these data inform future studies regarding cortical projection neuron subpopulations that are unique to primates and rodents, and indicate putative evolutionary relationships (Fame et al. 2017).

In this study, we have used CamKIIα-Cre recombinase mice, allowing us to identify GFP-expressing excitatory glutamatergic axons from the GABAergic inhibitory ones (Basu et al. 2008). Importantly, our results suggest that the long-projection axons in the corpus callosum could be efficiently distinguished using this method. The majority of callosal fibers connect cortical association areas containing both myelinated and nonmyelinated axons. To study their morphology, we immunolabeled them with MAG to determine the myelin sheath thickness between these axons (Trapp et al. 1989; Kursula et al. 2001). Collectively, the results, however supportive, could not resolve our questions.

In the traditional view of cortical physiology, communication between different cortical areas is thought to depend entirely on glutamatergic neurons. GABA-ergic cells act locally, within the area in which their somata are located. Thus, all IPSPs generated in the course of interaction between different cortical areas are believed to be generated disynaptically, after glutamatergic activation of local-circuit GABA-ergic neurons. Evidence has also shown that some GABAergic neurons of the perirhinal cortex participate in communication between different cortical rhinal areas (Apergis-Schoute et al. 2007). In view of this, we studied the ultrastructure of these long-projection callosal myelinated and nonmyelinated axons and found that the axon diameter of the excitatory axons differs according to their length and also upon myelination. Shorter nonmyelinated ones have smaller diameter than the longer and myelinated ones. In contrast, most of the inhibitory axons were myelinated and had a standard diameter with smaller variations. This contradicts the finding by some researchers that all long-range GABAergic axons have a larger diameter than glutamatergic excitatory axons. Their study was focused on long-range GABAergic projection neurons in the hippocampus, so other interareal GABAergic connections or long-projection callosal neurons connecting the 2 cerebral hemispheres might have different anatomical features. However, the overall thickness of myelination as well as thickness per stack was larger in inhibitory axons than the excitatory, which is supportive to the findings (Jinno et al. 2007).

Axonal diameter and myelinization has been measured in Rhesus monkeys at different parts of the corpus callosum (LaMantian and Rakic, 1990a). In adult monkeys the vast majority of callosal axons are myelinated (mean: ~75%) and showed regional differences in anterio-posterior direction (~72–96%). Our samples were taken from the anterior and the midline part of the corpus callosum. If there is no species difference in the spatial distribution of myelinated axons we predict that the inhibitory nonmyelinated axons are virtually nonexistent in the posterior part of the corpus callosum in mice.

Studies of conduction velocity of myelinated and nonmyelinated axons revealed that several key functional attributes depended on axon diameter and myelin sheath thickness (Fazan et al. 2005). Linear relationships between these 2 parameters were generally observed, although some researchers have reported a nonlinear relation among them (Sunderland and Roche 1958). Our simulation calculations showed a linear relationship, but interestingly the calculated conduction velocity and myelin thickness relationship was different for excitatory and inhibitory fibers.

Earlier reports predicted it is unlikely that myelinated fibers smaller than 0.2 mm could be found in any species based on theoretical and experimental bases (Waxman and Bennett 1972). The same report predicted that myelinated fibers with diameter between 0.2 and 1.0 mm conduct more rapidly than nonmyelinated ones with the same diameter. Our electron microscopy measurement showed that the diameter of all axons in mice corpus callosum were in this range. In addition our computational study also corroborated that myelinated axons have faster conduction velocity.

**Table 8:** Sensitivity of axonal conduction velocity to various biophysical parameters. These indices were evaluated and compared for axons of various lengths. Sensitivity was evaluated as the change in conduction velocity for relative change in the parameter.

**Parameter**	**Sensitivity**					
	**Excitatory myelinated**			**Inhibitory myelinated**		
	2 mm	12 mm	200 mm	2 mm	12 mm	200 mm
**Diameter**	0.39	0.29	0.28	0.33	0.28	0.27
**Axial resistivity**	−0.40	−0.30	−0.29	−0.35	−0.29	−0.29
**Lamella count**	0.47	0.43	0.42	0.43	0.39	0.39
**Lamella thickness**	0.47	0.43	0.42	0.44	0.39	0.39
**Internodal length**	−0.34	−0.39	−0.40	−0.28	−0.32	−0.33
**Internodal (cm)**	−0.51	−0.47	−0.47	−0.45	−0.42	−0.40

Myelination and node formation represent important developmental steps, which enable the axon to conduct impulses at a high speed, and to sustain rapidly propagating trains of impulses. CNS axons differ in diameter by nearly 100-fold (∼0.1–10 μm); therefore, they differ in cross-sectional area and volume by nearly 10,000-fold. Perge et al. (2012) studied functional requirements set and axon’s diameter surveying 16 fiber groups spanning nearly the full range of diameters in 5 species (guinea pig, rat, monkey, locust, and octopus). Their studies showed thin axons are most numerous. The mean firing frequencies, estimated for nine of the identified axon classes, were low for fibers with small diameter and high for larger ones, ranging from ∼1 to >100 Hz. They found that in order for axon to double its information transfer rate, it must at least double its firing rate. Hence, synchronization especially in the high frequency range of spatially distant areas would require large diameter axons with thick myelin sheets.

For methodological reasons we used transgenic mice to reveal differences between excitatory and inhibitory callosal axons. These experiments cannot be performed in human samples therefore the findings presented here need to be used with caution when translated to human brain functions. Functional lateralization is a fundamental principle of the human brain (Nowicka and Tacikowski 2011; Karolis et al. 2019) that is less explored in rodents. The lateralization in humans might be an evolutionary response to avoid excessive conduction delays between the hemispheres, which is a consequence of brain size expansion. Our data support this assumption because we showed that cross-hemisphere synchronization is limited due to conduction delays. In addition, based on recent studies using electron microscopy, diffusion tensor imaging and functional magnetic imaging techniques showing human callosal fiber functional and anatomical diversity (Yang et al. 2022, Häberling et al. 2011) it can be expected that human the callosal fiber composition, degree of myelination and the ratio of excitatory and inhibitory long-range callosal axons will show differences in the anterio-posterior axis of the corpus callosum.

Although there might be a sampling bias in our method (i.e. virus uptake or GFP/RFP expression of myelinated neurons differs from nonmyelinated ones) our finding that more nonmyelinated excitatory axons run in the corpus callosum than myelinated suggest a functional segregation of myelinated versus non/myelinated axon population. The prediction is that myelinated axons are responsible for cross-hemisphere synchronization, the information traveling through nonmyelinated axons represent a fundamentally different modality. In contrast, inhibitory axons were almost always myelinated thus likely playing an important role in interareal synchronization. A recent paper has shown that the locally-projecting, relatively short-axon inhibitory interneurons are a major source of myelinated axons within cortical gray matter, in contrast to the myelin in white matter that forms almost exclusively on the axons of long-distance projecting excitatory neurons (Tomassy et al. 2014). In particular, interneuronal myelin appears to be confined to interneurons containing the protein parvalbumin (Micheva et al. 2016; Stedehouder and Kushner 2017). Thus, it is likely that the myelinated inhibitory axons in the corpus callosum that we have measured originate from parvalbumin-expressing interneurons. Since NOS expressing GABAergic neurons also have long-rojection axon arbor (Tomioka et al. 2005), it is also possible that nonmyelinated callosal GABAerg axons originate from NOS expressing neurons. Whether these neurons have extensive local axon collateral network beside the long-range projection similar to the hippocampal long-range projection neurons (Sik et al. 1994, 1995) still needs to be determined. We also have to note that our study dealt with interhemispheral projections and intrahemispheral long-projection axons were not investigated. However, the modeling technique employed in the current study is very generic and used for modeling neurons of various brain regions. The specifics of the model, such as the composition of ion channels, their distribution along the axons, the neuronal morphology all are expected to vary from region to region; the extent of these changes being variable. Significant variations in parameters such as axonal diameters are uncommon even across species, and even less so between different regions of the cortex within the same species. Axonal diameter and the extent of myelination are the primary factors by which axons can vary their conduction velocities (Olivares et al. 2001). In the present study, we have explored variations of all these parameters over a wide range, and how they can affect conduction velocity. By doing this, we have tried to arrive at theoretical upper and lower bounds for the propagation velocity of signals in these axons, under various scenarios. Whether Although it is possible that difference in myelination in inter- versus intrahemispheral myelination profile exists after exhaustive literature search we were unable to find any published data on this matter that gives us the confidence that our result can be generalized to axons running in the callosum and in the white matter fasciculi.

Our study also demonstrated that the long-projection axon’s myelination thickness was linearly dependent on its axon diameter. Interestingly, the relationship of myelination and axon diameter, while linear in both cases, was different for excitatory and inhibitory fibers. The functional explanation might lie in the importance of inhibitory neurons in network synchronization. Since a small number of inhibitory neurons are needed for network synchronization but their efficient information transfer is essential for the normal function of the network it is expected that all long-projection inhibitory axons are myelinated, and the myelin sheath thickness is larger compared with the excitatory axons with the same diameter.

**Fig. 6:**
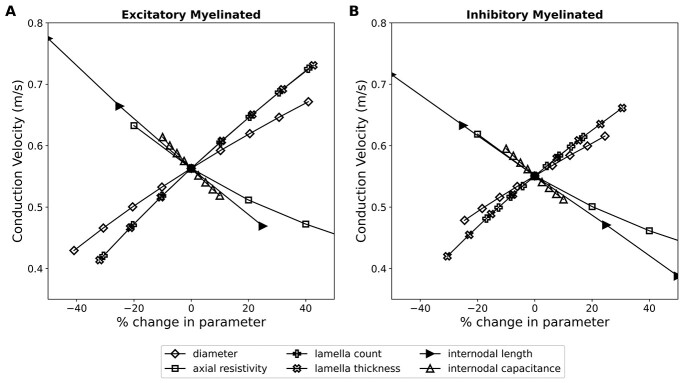
Comparison of sensitivity to changes in various biophysical parameters for both excitatory and inhibitory myelinated axons evaluated for axonal length of 12 mm.

**Fig. 7:**
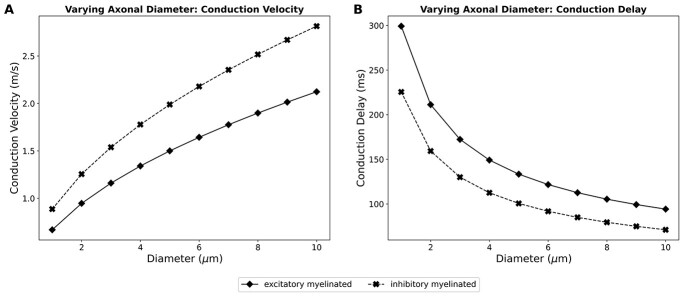
Simulation outcomes from varying the axonal diameter across a much larger range of values for both excitatory and inhibitory myelinated axons of length 200 mm A) shows the conduction velocity with increasing axonal diameters, B) shows the conduction delay from the soma to the end of the axon.

Although our finding that the mean myelin thickness of excitatory axons is much larger than the inhibitory seems in contradiction to previous reports on the hippocampus (Jinno et al. 2007), it is on closer consideration consistent with it. This is indicated in the smaller *g* ratio number (smaller *g* number indicates thicker myelination for a given axon diameter) for inhibitory compared with the excitatory axons. Previous studies have suggested that the practically feasible ways of increasing conduction velocity to compensate for larger brain sizes in a biological system, is via myelination or increasing the axon diameter. The majority of axon diameters remain more or less similar across species (~ 0.1–0.2 μm). A small minority of axons were found to have increasingly larger diameters (thereby larger conduction velocities) with increase in brain size. However, the increase in diameters does not sufficiently compensate for the delay in interhemispheric transmission time that accompanies larger brain sizes. Such biophysical constraints on the conduction velocity in larger brain sizes might have favored the development of hemispheric asymmetry, i.e. functional lateralization. Studies from our simulations illustrate how the various biophysical parameters affect axonal conduction. The various parameters influence the conduction velocities to varying extents, and even in the nature of these effects. The model built here was based on data obtained experimentally from mice, and thus it most accurately can be described as a mouse model for callosal axons. Nevertheless, we have additionally made a tentative attempt to utilize this model to simulate for larger brain sizes, and thereby extrapolate data as applicable to the human brain. Studies in the past have proposed maximal conduction distances in the human brain ~180 mm (Ringo et al. 1994). We have accordingly chosen an upper limit of 200 mm for our extrapolations. As described earlier, the base model produced a conduction delay of ~ 360 ms for the fastest fibers. Despite all the parameter variations that we explored, the overall conduction delay from the soma to the axon terminal could not be brought below 200 ms. Based on prior reports, which indicated that a small number of axons were found to have increasingly larger diameters in larger sized brains, we attempted to explore the effect of increasing the axonal diameters across a much wider range. The maximum axon diameters reported for callosal axons in the human brain lie between 9 and 11 μm (Aboitiz et al. 1992; Liewald et al. 2014). Accordingly, we explored the variation of axonal diameter between 1 and 10 μm. Our simulation findings are shown in Fig. 7. We observed a maximum conduction velocity of 2.81 m/s for the inhibitory myelinated axons, and 2.12 m/s for the excitatory myelinated axons. These translate to conduction times of 71.1 and 94.2 ms, respectively, for propagating an action potential from the soma to the axon terminal. Despite these large enhancements in the axonal diameters, the resultant conduction times do not seem to be sufficient to produce synchrony between the 2 hemispheres. Thus, our findings align with the prevailing notion that such biophysical constraints might have favored the development of functional lateralization in larger brains. The functional consequences of the conduction velocity differences caused by variations in myelination and axon diameter would be that slow transmitting nonmyelinated axons are unlikely to convey information across large distances via spike timing rather through firing rate, in line with a previous study (Wang et al. 2008).

The ultrastructural difference of myelin layers between excitatory and inhibitory axons was intriguing. Previous studies have demonstrated the heterogeneity of oligodendrocytes based on their morphology (Del Rio-Hortega 1928; Murtie et al. 2007; Vinet et al. 2010), gene expression (Marques et al. 2018), or myelinogenic potentials (Chong et al. 2012). A recent report by Zonouzi and coworkers showed that inhibitory and excitatory axons are myelinated by a different subset of oligodendrocytes (Zonouzi et al. 2019). Our data agree with this finding and even suggests that the 2 subsets of oligodendrocytes have a different biochemical mechanism to form a differently organized myelin sheets around the 2 subpopulation of axons.
